# Understanding beneficial self-management support and the meaning of user involvement in lifestyle interventions: a qualitative study from the perspective of healthcare professionals

**DOI:** 10.1186/s12913-020-4951-y

**Published:** 2020-02-05

**Authors:** Elin Salemonsen, Georg Førland, Britt Sætre Hansen, Anne Lise Holm

**Affiliations:** 1 Department of Health and Caring Science, Western Norway University of Applied Sciences, Faculty of Health and Social Sciences, Bjørnsons gate 45, 5528 Haugesund, Norway; 2 University of Stavanger, Faculty of Health Sciences, Kjell Arholmsgate 39, 4021 Stavanger, Norway

**Keywords:** Dialogue, Dignity, Healthcare professionals, Overweight and obesity, Primary care, Partnership, Self-management support, Shared responsibility, User involvement

## Abstract

**Background:**

In light of the high prevalence of overweight and obesity among adults and the subsequent stigmatization and health consequences, there is a need to develop effective interventions to support lifestyle change. The literature supports the key role of healthcare professionals (HPs) in facilitating self-management through lifestyle interventions for those with chronic conditions. However, there is a lack of knowledge about how HPs practice self-management support (SMS) and user involvement for persons afflicted by overweight or obesity in lifestyle interventions in primary care Healthy Life Centres (HLC). The aim of this study was to explore how HPs provide SMS and what user involvement implies for HPs in HLCs.

**Methods:**

An interpretative exploratory design, using qualitative thematic analysis of data from two focus group interviews with ten HPs from eight different HLCs, was conducted.

**Results:**

The analysis resulted in one overall theme; A partnership based on ethical awareness, non-judgemental attitude, dialogue and shared responsibility, comprising four interrelated themes: 1) Supporting self-efficacy, self-worth and dignity through an attitude of respect, acknowledgement and generosity, 2) Promoting self-belief and self-perceived health, 3) Collaborating and sharing responsibility, and 4) Being flexible, adjusting and sharing time.

**Conclusion:**

HPs in HLCs see service users as equal partners in a collaboration based on shared responsibility, acknowledgement and generosity. In order to help, their practice involves a heightened level of ethical awareness, including a non-judgemental attitude and dialogue. HPs in HLCs have something to teach us about ethical acting and helping persons who are struggling with overweight or obesity to change their lifestyle and regain dignity. They seem to see the service users’ existential needs and have learned the art of meeting the other in her/his most vulnerable situation i.e., seeking help for a “wrong lifestyle”. It may be time to highlight the need for SMS and user involvement to focus on shared responsibility in partnership rather than personal responsibility. More research is required to explore the conditions for such practice.

## Background

Overweight and obesity are complex conditions with serious social and psychological dimensions and one of the contributing factors of non-communicable diseases (NCDs), including type 2 diabetes, cardiovascular diseases, chronic respiratory conditions and cancer [1, 2]. Lifestyle changes are difficult and long-term weight management is associated with emotional distress and a high risk of failure [3, 4]. Shame and stigma can be a barrier to seeking help for weight management [5–7] and people with internalized stigma tend to have a lower self-worth [8].

The increasing number of patients with chronic diseases represents a challenge for the healthcare system and has led to an increase in the development of educational self-management interventions [9–12]. There is a growing interest in the impact and outcomes of self-management interventions [13], and the literature supports the key role of healthcare professionals (HPs) in facilitating self-management in chronic conditions and lifestyle interventions [14, 15]. These self-management support (SMS) interventions aim to equip service users and patients with the necessary information and skills to manage their own healthcare (independency), maintain optimal health and minimize the consequences of their conditions [9, 10, 12, 16]. Self-management is defined as an individual’s ability to detect and manage symptoms, treatment, physical and psychosocial consequences, as well as the lifestyle changes (such as exercise and diet) inherent in living with a chronic condition [11, 17]. One of the key goal of SMS is to raise self-efficacy [10, 12], the belief of individuals in their own ability to manage different tasks [18]. SMS approaches emphasize a clinical partnership, collaborative care, promote service users’ identification and achievement of realistic goals and teach problem-solving skills [10, 19]. Potential benefits of SMS include quality care tailored to the service users’ preferences and situation [17], which in some cases improve outcomes and reduce costs [10, 13].

There has been an increased commitment in health policies to empower and more actively involve patients in their healthcare through a bottom-up approach [20–23]. The intended consequences of user involvement include heightening people’s level of independence, with the objectives of enabling greater equality and more democratic decision-making [24]. Additionally, user involvement is seen as a means of ensuring accountability and balancing professional power, as well as improved health services and quality of care [22, 25]. According to Beresford [24], user involvement is a term which is poorly defined and carelessly used. User involvement is often treated in isolation as a technical rather than an ideological matter and needs to be understood in the historical, political, ideological and cultural context [24]. In this study, user involvement is understood as a clinical partnership between the service user and HPs [22], and characterised in terms of co-production of healthcare service [20, 21].

As a part of Norway’s national strategy to prevent NCDs, improve health and reduce morbidity, Healthy Life Centres (HLCs) have been established as part of the municipalities’ primary healthcare system [16, 26]. The purpose of HLCs is to support lifestyle change and promote self-management. The interventions offered are based on a salutogenic foundation, using motivational interview (MI) as one of the conversational approaches [16]. MI is a directed, person-centred counselling style that involves users and elicits behaviour change. It is defined by its spirit as a facilitative style for interpersonal relationship [27, 28]. The underlying spirit of MI, its mind-set or perspective on how to practise it is important, emphasizing four interrelated elements; partnership (collaboration), acceptance, compassion and evocation [28]. MI is also described being based on the principles of experimental social psychology and the concept of self-efficacy [29].

Previous studies from the service user perspective reveal that user involvement is significant for the quality of the healthcare service in HLCs and highlight acknowledgement and individualized SMS [30, 31]. One qualitative study from a HLC found that having a trustful relationship with the providers, being respected and experiencing continuity in the care were essential for service user involvement [30]. The support from significant others, peers, family, friends and health professionals is important for self-management and individual empowerment [3, 30–32]. Long-term self-worth support is essential for starting, continuing and participating in lifestyle change processes and a means to self-management [31].

From HPs’ perspective, one qualitative study by Abildsnes et al. [33] found that HPs emphasized person-centred advice based on the participants’ willingness to change and their impression of the participants’ condition and life circumstances. Another qualitative study by Sagsveen et al. [34] explored how HPs described involving service users in individual- and group-based counselling and activities at HLCs. It demonstrates the importance of HPs building a trustful relationship, adjusting to the users’ needs, strengthening the users’ ownership of and participation in the lifestyle change process and that HPs are involving users through MI. Sagsveen et al. [34] call for greater reflection on what user involvement implies in the HLC and in each user’s situation.

There is a need for more knowledge of HPs’ experience and perceptions in order to understand how they can provide a qualitatively good healthcare service for persons afflicted by overweight or obesity. There is also a need to better understand how HPs create a mutual relationship (partnership) with service users, practise SMS, promote self-management and what user involvement implies for HPs in HLCs. Due to the paucity of studies pertaining to the perspective of HPs in HLCs, the aim of this study was to explore how HPs provide SMS and what user involvement implies for HPs in HLCs.

## Method

### Design

Qualitative methodologies aim to explore complex phenomena of human experiences, meaning and attitudes [35, 36]. An interpretative exploratory design was chosen in order to gain a deeper understanding of beneficial SMS and user involvement as described by HPs working in HLCs. Focus groups are a suitable method for data collection [37, 38]. In this study data were collected by means of two focus group interviews, collecting data through group interaction and discussion on a topic determined by the researcher [38].

### Study context

The HLC is an interdisciplinary primary healthcare service, which offers individual and group-based lifestyle interventions for people at risk of NCDs or in need of support to change their lifestyle or manage chronic conditions [16]. The initial health conversation is based on each service user’s needs and desire for help, after which a group-based healthy diet course and/or physical activity sessions was offered. If desired, individual counselling is also available. Group-based healthy diet courses consist of four to five two-hour sessions with theory and practical tasks. Physical activity sessions, two to three times a week, are based on both indoor-and outdoor activities. The purpose of HLCs is to promote health and empower service users to engage in better self-management. HLCs are easily accessible for service users through direct contact or by referrals from general practioners (GPs). The lifestyle interventions that are provided by HPs (including public health nurses, psychiatric nurses, physiotherapists, dietitians and bachelor’s in public health) employ a person-centred approach and use e.g. MI as a conversational method. An intervention lasts for 3 months with the possibility to extend it on two occasions. The practice of extending participation and the organisation of the HLCs differs between the various municipalities. Small communities often have inter-municipal collaboration [16].

### Participants and recruitment

The participants for this focus group study were recruited from different HLCs in Western Norway, and 15 HLCs was invited by e-mail to participate. The aim was to recruit HPs with experience from lifestyle interventions in HLCs working with people afflicted by overweight and obesity. Purposive sampling [39, 40] was used to establish focus groups with variation in terms of occupational background, from well-established and new HLCs as well as urban and rural, small and medium-sized municipalities. Ten HPs (nine women and one man, aged 26 to 49 years) from eight different HLCs participated in two focus groups (Table 1). Information power guided the sample size [39].

**Table 1:** Characteristics of HLCs and HPs in the two focus groups

	Occupational background	Gender	Years of clinical experience (HLCs)	Rural /Urban	Population	Years of HLC establishment	Number of employees
Focus group 1 (FG-1)	Physiotherapists (2), psychiatric nurse (1) and public health nurse (1)	Female (3) Male (1)	1–7	Urban (2) Rural (2)	8.500- 38.000	2–5	1–2
Focus group 2 (FG-2)	Physiotherapists (4), bachelor’s in public health (1) and nutritionist (1)	Female (6)	2–7	Urban (3) Rural (1)	12.000–19.500	2–7	1–4

### Data collection

Focus group interviews are suitable for exploring new areas with sparse knowledge [37], and to explore experiences, attitude and views [38, 41]. Focus groups were employed to collect the qualitative data in this study [37–39]. The characteristic of focus groups (FG) and group interviews as research method and data collection method is their explicit use of group interaction and discussions to produce data and insights that would be less accessible without the interaction found in a group [38].

Participation was voluntary and everyone received both an oral and a written invitation and information about the study prior to the interviews. The focus group interviews took place at one university campus and one local HLC in 2017, based on practical considerations such as the shortest possible travel distance for the participants. In accordance with an explorative design [42], a flexible format topic guide (Table 2) with loosely phrased questions was developed to guide the group discussions [37, 38]. The moderator and first author (ES) presented her background as public health nurse as well as academic and clinical interests. The first author (ES) moderated the discussions, and added supplementary open- ended question when necessary. The co-moderator made notes and observed the interaction and dynamics in the group [38]. Most of the participants had met before and several of them collaborated in an inter-municipal cooperation network. The form of the focus group interview was open and the participants were invited to speak freely about their experiences of work in the HLC. The participants played an active part in the discussions in a highly reflective manner and the familiarity from previous networks and working together seems to make them feel comfortable. Each focus group interview lasted 120 min, was recorded on audio-files and subsequently transcribed.

**Table 2:** Topic guide in focus group interviews

Self-management support (SMS):	
• What do you experience as beneficial help and support for the service users afflicted by overweight or obesity attending lifestyle interventions in the HLCs?	
• What do you perceive as beneficial support for lifestyle change?	
• How do the service users describe beneficial support?	
• How do you promote self-management in the interventions?	
• Can you describe how you work?	
• What is important in the promotion of self-management and supporting lifestyle change?	
User involvement:	
• What do you understand by user involvement at the HLCs?	
• What is important in the involvement of the service users?	
• How do you involve the users in the intervention and in the process of change?	
• What is the significance of user involvement?	
• What do user involvement imply?	

### Data analysis

Thematic analysis as described by Braun and Clarke [43] and Vaismoradi et al. [36] was used to analyse the data from the focus groups. Thematic analysis is a method to identify, analyse and report patterns and themes in qualitative data [36, 43]. The aim is to provide description of both the manifest (semantic, explicit) and latent content (underlying interpretative level), pattern response or meaning in the text to develop a new understanding of the phenomenon under study and to answer the research question [43]. The theoretical framework was grounded in an inductive text-driven search for patterns described by Krippendorf [44]. The approach in the interpretation and theme development was abductive in the form of a hermeneutical spiral [42, 45, 46].

The transcripts from the two focus group interviews with HPs were read independently by all the authors (ES, GF, BSH & ALH). Patterns and themes identified in the data were coded, discussed in group meetings, refined further and organized into themes. A matrix was developed by the first author (ES) and all data were systematically apportioned using Excel and tables. Related text elements were reassembled in a new matrix, abstracted and grouped into themes, which were discussed by all the authors in the context of our aim and research question. Data were analysed within each of the focus groups and across the groups to identify both common and specific themes. The primary analysis, coding (data-reduction relevant for the research question) and categorization of the meaning units and preliminary themes were performed by ES. In addition, the themes were discussed, revised and interpreted into one overall theme by all the authors. Theme development was conducted in an analytic cyclical process (hermeneutic spiral [46], both inductive and abductive [44, 45]). Labelling the themes and overall theme with a phrase or sentence was preferable to a single word label for capturing complete ideas [47] or something important in relation to the overall research question [43]. The selection of quotations to illustrate the data was performed by ES.

## Results

The analysis resulted in one overall theme; A partnership based on ethical awareness, non-judgemental attitude, dialogue and shared responsibility, comprising four interrelated themes: 1) Supporting self-efficacy, self-worth and dignity through an attitude of respect, acknowledgement and generosity, 2) Promoting self-belief and self-perceived health, 3) Collaborating and sharing responsibility, and 4) Being flexible, adjusting and sharing time (Table 3).

**Table 3:** Overall theme and themes describing how HPs provide SMS and what user involvement implies for HPs in HLCs

Overall Theme	A partnership based on ethical awareness, non-judgemental attitude, dialogue and shared responsibility			
Theme	Supporting self-efficacy, self-worth and dignity through an attitude of respect, acknowledgement and generosity	Promoting self-belief and self-perceived health	Collaborating and sharing responsibility	Being flexible, adjusting and sharing time

### A partnership based on ethical awareness, non-judgemental attitude, dialogue and shared responsibility

HPs provide SMS and user involvement in lifestyle interventions in HLCs through ethical awareness, a non-judgemental and open attitude and dialogue. Self-efficacy, self-worth and dignity are supported by a respectful way of being, acknowledging the service users for who they are. HPs aim to prevent new disappointments and promote self-belief and better perceived health to support self-management. User involvement and SMS takes place through shared responsibility in a partnership with the service users. HPs take responsibility for creating a mutual and trustful relationship, emphasizing equality, acknowledgement and generosity in this collaborative partnership. Flexibility and adjustment of the support to the service users’ needs and situation are essential, and the temporal nature of the collaborative partnership and follow-up is important.

### Supporting self-efficacy, self-worth and dignity through an attitude of respect, acknowledgement and generosity

The first theme described how HPs supported self-efficacy, self-worth and dignity through an open, positive and accepting attitude and professionalism, communicating generosity and acknowledgement to promote self-management. HPs described user involvement as a way of being, emphasizing human values and generosity, using humour and sharing personal experiences. They wanted to be a helpful partner who is sensitive, attentive, curious and genuinely interested in the participants as persons. It was important for them to meet the service users were they were with friendliness and hospitality, to see, listen and acknowledge the service users for who they are:

*The most important is to be interested in the person in front of you and to explore: “what is important for you in your life and what would you like me to help you with?”* (10-FG2).

The initial health conversation was important and they described using MI or other health pedagogic conversations tools. HPs underlined the necessity of creating a relationship and employing their communication skills. They described their attempts to create an environment that invited confidential conversations, opened up for questions and enabled the service users to dare to tell their story to someone with time to listen:

*Caring and communication skills are essential.* (4-FG1).

HPs expressed a perception of service users as experts on themselves, possessing complementary expertise in the change process and the necessity of withholding their own opinion of the service users’ needs and what she/he should do. All of them described the importance of meeting the service users with respect, seeing them as valuable persons and addressing their guilt, shame, defeats and relapses by means of normalisation and humanisation, in an attempt to enable them to regain self-belief:

*We never tell them what to do or not to do (6-FG2). We do not moralize or be condescending* (7-FG2) … *and we ask for permission to give them advice.* (8-FG2).

Several of the HPs gave examples for how they asked for permission to give advice, such as kindly asking:

*“Would you like me to tell you what has helped others?* or *“Would you like me to tell you what we have experienced as helpful?”* (5-FG2).

HPs noticed the importance of giving feedback to and expressing belief in service users, their capability, strength and power, to support self-efficacy. It was also essential to express an understanding of the challenges of lifestyle change that involves more than “to exercise more and eat less” and to avoid being patronizing so that service users do not have to defend themselves. HPs explained that the concept of HLCs is that HPs respect each service users own reasons for seeking help there. HPs experienced that service users appreciated their kindness and lack of strictness, their acknowledgement despite failures, their non-judgemental attitude, the absence of condescending behaviour and not making service users feel that they have been given up on managing lifestyle change:

*We experience the service users’ comfort and thankfulness for arriving in an arena where they do not need to explain or justify why they need to change their lifestyle, where there is nobody to arrest or judge them.* (9-FG2).

### Promoting self-belief and self-perceived health

The second theme described how HPs aim to guide and promote self-management and user involvement by strengthening service users’ belief in themselves and avoiding new disappointments. An important purpose of HLCs is to improve service users’ self-perceived health. The initial health conversation lays the foundation for a trustful relationship where the HP becomes a helpful partner. HPs recognized that the service users had complex challenges that made it difficult for them to change their dietary and exercise habits. Some of the most important issues described by HPs were the need to emphasize well-being, help the service users to thrive and make them want to come back and continue to participate in the HLC intervention:

*Our greatest goal is to keep them, make them thrive and give them a feeling of meaningfulness by participating in the HLC.* (1-FG1).

HPs described that some of the service users had expected to be given a fixed plan and that they had to turn this expectation into an understanding of the importance of service users making the plan themselves. They helped the service users to set realistic goals that were possible to reach, avoid new disappointments, failure and setback and helped them get back on track when they had a relapse. They believed that the service users needed to achieve some goals in order to regain belief in themselves. Several of the HPs recognized getting better at assessing and identifying service users who were not yet ready to start a full lifestyle intervention due to their excessive distress in life, with the intention of avoiding new disappointments:

*I have become better at questioning the service users who have too much psychological distress and life-challenges if this is the right time to start a lifestyle change.* (4-FG1).

HPs recognized the necessity of adopting a holistic approach and addressing psychological challenges and emotional distress. They described working with “life-self-management” as essential rather than only covering what to eat and the amount of exercise and activity:

*I believe that this group of people have complex conditions and challenges and need help to manage life, not only advice about what to eat or how much to exercise.* (7- FG2).

HPs also described trying to turn the focus on better perceived health away from weight, BMI, special diets and slimming:

*Our job is to strengthen the service users to take care of their own health, by explaining and emphasizing that this is not a slimming programme.* (2-FG1).

They noticed that the service users were motivated by experiencing the effects of training. According to the HPs, the service users reported having more energy, finding it easier to perform daily activity, greater well-being, more confidence, increased self-efficacy and motivation after participating in lifestyle interventions in HLCs. For several of the service users this could include better fitness, feeling stronger, lower blood pressure, blood glucose and cholesterol, while some of them also mentioned weight reduction. A number of the HPs stated that the purpose of user involvement is for the service users to discover their own resources, the significance of good health and strength and increase their self-efficacy. HPs emphasized the need to focus on the service users’ resources, give them feedback and help them to see all the small changes they had achieved. Regular follow-up conversations were often necessary to make them aware of what they managed and to increase positive self-talk. They experienced that positive feedback on achievements led to self-belief, pride and motivation:

*They need feedback on every little achievement so that they don’t give up. They need to be conscious of the small changes they make…* (8-FG). *Much of the purpose of user involvement is to let the service users discover which possibilities and resources they have and raise their belief in self-management.* (3-FG1).

### Collaborating and sharing responsibility

The third theme described how HPs emphasized collaboration and partnership with the service users as well as the importance of a trustful relationship and shared responsibility to increase user involvement and self-management. HPs outlined how they explored the service users’ needs, set goals together and sometimes did the problem solving together with the service users. They experienced that it was more helpful to be a partner who listens than an expert who gives advice. HPs emphasized equality and the value of complementary competence. They described a philosophy based on the importance of the service users’ experiential knowledge for all parties:

*They are the ones who know where the shoe pinches, they are the experts on themselves and I think that we have complementary expertise.* (7-FG2).

They stated that service users and HPs had different responsibilities. The users’ responsibility was to follow the plan they were involved in making, participate and attend all appointments and intervention sessions, while the HPs were responsible for letting the service users’ voices be heard, being available, addressing expectations, helping, being generous, interested and providing follow-up. Written contracts signed by both parties outlined the expectations, responsibilities and commitments. Relational commitments and expectations to participate were important, but there were no commitments or expectations about outcome or weight loss. The relational commitments, meaning that someone is waiting for you (also in groups), are both desirable and important for both parties:

*We write and sign a contract that we have to adhere to. We make commitments to provide follow-up, while the service users make commitments to follow their plan … and the service users appreciate the commitments and expectations because of the difficulties getting into the activity groups and “getting started” on their own.* (10- FG2).

### Being flexible, adjusting and sharing time

The fourth and last theme described the importance of adjusting self-management support to the service users’ needs and the significance of time, flexibility and extended support for lifestyle change. HPs described their role as being an available, supportive partner in the process of change, guiding each service user in the best possible way. They stated that sufficient time to get to know the service users and their values in order to identify their needs creates the basis for user involvement, as well as for the possibility to adjust and tailor the person-centred care and individualized support. Giving the service users time to tell their story and exploring their concerns (not the experts’ concerns) were essential. They acknowledged that they had more time for exploring the service users’ needs and values compared to GPs:

*It is important to have time to listen and to get to know the person in front of you, who feel the challenges in her/his body* (9-FG2).

HPs described the service users as a very heterogeneous group with different resources, needs and wishes. Several of them had complex challenges and different follow-up needs. HPs recognized the importance of supporting the service users’ own choices and goals, not those of the “experts” or professionals:

*“We let them define their own goals and help them to make a plan”* (7-FG2).

The HPs described that being flexible and adjusting within the limits of what was possible was required. It was necessary to be sensitive, give service users an opportunity for expressing freely and listening to them, even if the HPs could not fulfil all their wishes. It was important to emphasize well-being and offer an intervention with a variation in activities that was meaningful and related to service users’ needs and wishes. HPs described their contribution to creating new structures, routines and habits, highlighting the transferability to the service users’ situation and everyday life. In this process of adjustment, HPs tried to avoid giving advice too quickly. They attempted to “lay back” and let the service users find the solutions on their own and use their problem-solving skills first, describing this as both time-consuming and crucial. Communication skills were perceived as more important than having the right answer to every question:

*It is important to learn to lean back and let the service users be in control.* (9-FG2).

Supervised group-sessions and individual health conversations were recognized as helpful for both parties. In activity group-sessions, HPs regularly experienced beneficial contact and an opportunity for follow-up. Individuality was perceived as a possibility, even in group-sessions, by creating a safe environment for the acceptance of diversity and that everyone and everything was “good enough”. Flexibility related to the service users’ preference for individual counselling and support, which was accepted and possible, although some of the HPs tried to give the service users a friendly push toward group participation. Time was essential in this process of “persuasion” to convince the service users of the advantages of group participation. However, they accepted those who absolutely did not want to “belong” to a group without compromising their healthcare:

*The great thing is that we have both groups and individual support. Those who do not want to be in a group can have individual follow-up, which is perfectly ok.* (9- FG2).

They described trying to make the service users understand that lifestyle change takes time by communicating a long-term perspective and stressing that change does not occur in 2 weeks. They tried to confirm the normality of ups and downs, trial and error, and the possibility to get back on track. HPs perceived long-term follow-up as one of the most important conditions for successful lifestyle change. Several service users repeatedly joined a new course. HPs allowed those who needed extended follow-up to continue after the end of an intervention period and “gaming the system” of a maximum of three 3-month interventions. However, this was practised differently in the various HLCs:

*There is something about recognizing that change takes time and we don’t expect the service users to achieve their goals of change by the end of the course.* (4-FG1) *We let them continue with the training sessions after the end of the intervention.* (5- FG2).

## Discussion

The aim of this study was to explore how HPs provide SMS and what user involvement implies for the HPs in HLCs. The HPs in this study exhibited a high degree of self-reflection and an in-depth understanding of human needs and behaviour when discussing their role as supervisors responsible for SMS and user involvement for persons afflicted by overweight or obesity. The overall findings suggest that a partnership based on ethical awareness, non-judgemental attitude and dialogue as well as shared responsibility is a description of how HPs provide SMS and involve service users in the lifestyle interventions in the HLC. This discussion will focus on the overall theme*; A partnership based on ethical awareness, non-judgemental attitude, dialogue and shared responsibility*. We will discussed the elements in this theme in light of previous studies and the literature.

*A partnership based on ethical awareness,* shows that the SMS in HLCs takes place through a trustful relationship (partnership) with the service users. The HPs described both relational and communicational skills as essential for SMS and user involvement. This is in line with the study by Sagsveen et al. [34], underpinning the importance of participative communication skills among HPs in HLCs to promote involvement. The HPs in our study emphasized human values and generosity as the core relational skills, in addition to the importance of being genuinely interested, curious, sensitive, friendly and helpful. This attitude can be seen as a means to get to know each service user and understand her/his values, needs and interests. This awareness also reflects the ethical principles of autonomy and beneficence [48]. The importance of a trustful relationship has been highlighted in previous studies [30, 34, 49–51]. Our findings add to the literature by underlining the importance of a respectful way of being for building a trustful relationship with service users that places HPs in a position to help with overweight or obesity. There is a need to strengthen the service users’ experience of dignity and self-worth due to their social stigma that goes beyond their self-worth [4]. The service users’ shame and search for dignity [4] imply an ethical requirement for HPs in HLCs to meet these service users’ existential need for integrity and dignity, and it seems as if the HPs in the present study are doing just that. Our study indicates that HPs are meeting the service users’ existential needs with self-worth support, acknowledging them for who they are and being genuinely interested in them. Their descriptions illustrate how the HPs managed to be sensitive and meet their perceived “wrong” lifestyle, vulnerability and shame with respect, acknowledgement and generosity, allowing them to fail and not being condescending about their lifestyle. According to Gjengedal et al. [52], being sensitive to the vulnerability of the other may be a key to acting ethically. The HPs in our study saw “service users as experts on themselves”, which may imply safeguarding their autonomy and taking advantage of their own contribution so that they can preserve something of themselves and regain their dignity. In the findings from Salemonsen et al. [31], the service users in HLCs highlighted the professionals’ competence, attitude and the feeling of increased self-worth and dignity they obtained through participation in the HLCs. Acceptance and self-worth support may lead to a positive body image and less guilt and shame. According to Tranvåg et al. [53], confirming the person’s worthiness and sense of self involves genuine respect for each individual as a unique human being and such confirmation is an essential prerequisite for autonomy and integrity.

*A non-judgemental attitude and dialogue,* is revealed by the findings in our study, describing HPs using elements of MI in their dialogue with the service users. Partnership in MI means an active collaboration between experts, with a view that people are the undisputed experts on themselves. Acceptance in MI includes four aspects of absolute worth, accurate empathy, autonomy support and affirmation [27, 28]. It seems as if the HPs in our study were influenced by MI and made their own dynamic “tool-box” of elements that they experience as beneficial. Their use of elements, adjustment and the adaptive capacity of this communication style shows their competence and that the spirit of MI and humanistic values have become an integrated part of their thinking and way of working. The adaptive capacity and use of elements may characterize a professional who has integrated these into her/his way of doing, being and meeting the “other”, described by Benner [54] as a theory from novice to expert in nursing practice. In addition, the findings in our study confirm that the HPs emphasize a person-centred approach found in Rogers’ [55] theory of a client centred approach in psychological therapy and Buber’s [56] theory of dialogue. Rogers highlights the importance of genuineness, creating a climate for change through acceptance and caring, emphatic understanding and listening, extending unconditional positive regard [55]. The philosophy of Buber and his theory of dialogue emphasizes an “I and Thou” approach in the conversation as opposed to an “I and It” approach. Buber states that the ontological basis of human existence lies in the dialogue between the self and others and that dialogue is about relationality and meetings between people [56]. In HLCs the non-judgemental attitude and dialogue seem to be an integrated part of the practice and personality of authentic and honest HPs. Their way of seeing and being demonstrate that they are firmly rooted in humanistic values that support existential needs. Consequently, the use of a non-judgemental dialogue and attitude, sensitivity and hospitality may lead to a wish to participate in the lifestyle interventions in HLCs and give service users a sense of worth and motivation for continuing lifestyle changes. Healthcare settings have been reported to be one of the sources of weight-stigma [57, 58]. Several HPs hold strong negative attitudes about people with obesity [5, 59], and this attitude and weight-stigma can reduce the quality of care and weight-management [5, 60]. We believe that HPs in general, in healthcare services, have something to learn from HPs in HLCs and their “MI –spirit”. Negative attitudes affect whether one has a non-judgemental attitude or not, and changing attitudes among HPs may be an important and necessary step to help persons in vulnerable situations.

*Shared responsibility,* shows how HPs taking responsibility for creating a mutual relationship through interaction and collaboration. They emphasized equality, in addition to the necessity of the service users’ experiential knowledge and complementary competence in this clinical partnership. A collaborative partnership is described in the literature as one of the most important prerequisites in SMS [10]. Additionally, the HPs communicated that the service users have no responsibility for the outcome or for weight loss. They emphasized participation and that the service users perceived better health, well-being and a healthier lifestyle. This shows that HPs are aware of their responsibility as professionals and assign responsibility to the service users for the purpose of sharing responsibility. Sharing responsibility may also reduce the pressure for weight-loss and the feeling of guilt and shame. In other studies, however, HPs held patients accountable for both their body weight and their attributed lack of responsibility for investment in change [60]. A dominance of a traditional model of care, where HPs remained in a position of authority and limited collaboration was found. The psychosocial and temporal nature of interaction was excluded and the context was characterized by the service users’ individual responsibility and accountability for self-management and adherence [61]. Those findings may challenge equality, respect and acknowledgement in the clinical SMS partnership, and are inconsistence with our findings.

The HPs in our study practice SMS through tailored support and counselling, emphasizing flexibility, adjustment and sharing time. The importance of flexibility and adjusting support to service users’ needs and context is supported by several previous studies that demonstrated how essential such aspects are for lifestyle change and self-management in chronic conditions [50, 51, 62, 63]. HPs saw the need for frequent support and follow-up over time for several of the service users. Previous studies emphasize follow-up as a prerequisite for maintenance of lifestyle change [50, 63, 64], maybe over several years [31, 65–67]. Establishing a trustful relationship takes time. The HPs are aware of this and take responsibility for prioritizing the allocation of time to get to know the service users and build a relationship. In a study exploring HPs’ perceptions of user involvement in HLCs, being present in the situation and devoting sufficient time to the health conversations were also described as essential [34]. HPs in our study made a decision to give those service users who needed extended follow-up more time and counselling than the intervention entailed. While this may be interpreted as a form of “gaming the system”, it can also be interpreted as HPs assuming their relational and moral responsibility for the service users’ need for extended help and support. So, what do relational and moral responsibility towards the service users imply? The HPs in our study described their responsibility to meet the service users with respect, hospitality and a desire to help the other, often asking them; *“what is important for you in your life”* and *“what would you like me to help you with?”.* They described a practice of moral responsibility and ethical awareness similar to our understanding of Levinas’ theory of responsibility. According to Morgan [68], Levinas teaches us to acknowledge what we owe to others, to be kind, caring and generous. Responsibility is about our commitment to take care of or deal with and that moral responsibility arises in the face-to-face interaction with another person. In all relationships, we are faced with a demand to take responsibility for the other and are thus not free to choose our moral responsibility. This ethical and moral responsibility cannot be shared or given away [68–70]. Being a responsible HP entails facing up to the consequences of our behaviour and actions. By prioritizing time and focusing on the service users’ needs and situation, it seems as if the Levinanian responsibility comes into play. In relation to time, continuity and having enough time to get to know the service users and their needs is important and in line with other studies [34, 49]. How HPs in HLCs manage to prioritize time and their challenges and needs related to the organisation of lifestyle interventions in HLCs requires further investigation.

The focus on individual responsibility for health in contemporary society described in earlier studies [71–74] shows that responsibility for behaviour change is often discussed at an individual level and rarely at a professional or societal level. Very few studies focus on shared responsibility between the partners in a clinical partnership in either lifestyle interventions or society in general. Consequently, this may reflect the major attitude towards individual responsibility in society, which may add more blame or shame to people afflicted by overweight or obesity. Our findings add to the literature and illustrate how ethical awareness, a non-judgemental attitude, shared responsibility and avoidance of negotiation of responsibility for outcome and weight management may strengthen the service users’ self-efficacy, self-worth and dignity.

### Trustworthiness

Trustworthiness in this qualitative study is based on Lincoln and Guba [75] and the aspects of credibility, dependability, confirmability and transferability. Confirmability and dependability of the research was confirmed through the systematic, analysis and discussion of the findings between all the researchers over a period of time [35, 37, 43, 75]. Quotations from the interview data have been included in order to illustrate and ensure the credibility and dependability of the HPs’ perspectives and descriptions. Data collection and context are carefully described in order for the reader to decide on the transferability of the findings to similar contexts. The interpretation was influenced by the preunderstanding of the researchers, which must be taken into account [46, 76]. The authors have various clinical experiences and disciplinary backgrounds such as public health nurse (ES & BSH), psychiatric nurse (ALH), patient education (GF) and intensive care (BSH), which enriched the analysis and interpretation, thereby increasing trustworthiness and minimizing potential bias. The present paper was cross-checked to comply with the consolidated criteria for reporting qualitative studies using the 32-item COREQ checklist [77].

### Strengths and limitations

Our study has contributed to a deeper understanding of HPs’ practice of SMS and service user involvement. Focus group interviews were a plausible method for discovering this knowledge of HPs’ values and reflexivity. HLCs are a relatively new healthcare service in primary care. Due to the sparse knowledge and understanding of the HPs’ perspective, this study contributes to deepening the understanding of how to provide a qualitatively good healthcare service. One possible limitation might be the gender balance with only one male participant. However, this reflects the general gender balance in HLCs, which have a majority of female employees. Another limitation that should also be taken into consideration concerns the composition of the focus groups. The participants in one of the groups had experience of inter-municipal collaboration over a period of several years, while the participants in the other group had only met a few times and had less experience of working in a HLC. However, none of the participants appeared to be reticent about expressing their opinions and perceptions. A potential limitation is related to the small number of focus groups, however information power guided the sample size [39].

## Conclusion

This study reveals that HPs in HLCs provide SMS and involve service users through extensive tailored support based on the service users’ needs and situation. The findings show that the HPs see the service users as equal partners in a collaborative partnership based on shared responsibility, acknowledgement and generosity. To be in a position to help, their practice involves a heightened level of ethical awareness, including a non-judgemental attitude and dialogue. The HPs seems to be dedicated and to take a personal interest in those seeking help through openness, compassion, sensitivity and a positive attitude. HPs in HLCs have something to teach us when it comes to ethical acting and helping persons who are struggling with overweight or obesity to change their lifestyle and regain dignity. They appear to see the service users’ existential needs and have learned the art of meeting the “other” in one of her/his most vulnerable situations i.e., seeking help for a “wrong lifestyle”. Our findings contribute to a wider understanding of user involvement and SMS in lifestyle change. It may be time to highlight the need for SMS and user involvement to focus on shared responsibility in partnership rather than personal responsibility. More research is required to explore the conditions for such practice.

## Data Availability

The dataset used and analysed during the current study are available from the corresponding author on reasonable request.

## Abbreviations

FG: Focus group
GPs: General practioners
HLCs: Healthy Life Centres
HPs: Health professionals
MI: Motivational interview
NCDs: Non-communicable diseases
SMS: Self-management support

## References

[1] World Health Organization (2019). Controlling the global obesity epidemic.

[2] World Health Organization (2018). Noncommunicable diseases Fact sheet.

[3] Følling IS, Solbjør M, Helvik A-S (2015). Previous experiences and emotional baggage as barriers to lifestyle change - a qualitative study of Norwegian healthy life Centre participants. BMC Fam Pract.

[4] Salemonsen E, Hansen BS, Førland G, Holm AL (2018). Healthy life Centre participants’ perceptions of living with overweight or obesity and seeking help for a perceived “wrong” lifestyle - a qualitative interview study. BMC Obesity.

[5] Phelan SM, Burgess DJ, Yeazel MW, Hellerstedt WL, Griffin JM, van Ryn M (2015). Impact of weight bias and stigma on quality of care and outcomes for patients with obesity. Obes Rev.

[6] Spooner C, Jayasinghe UW, Faruqi N, Stocks N, Harris MF (2018). Predictors of weight stigma experienced by middle-older aged, general-practice patients with obesity in disadvantaged areas of Australia: a cross-sectional study. BMC Public Health.

[7] Puhl RM, Quinn DM, Weisz BM, Suh YJ (2017). The role of stigma in weight loss maintenance among US adults. Ann Behav Med.

[8] Kato A, Fujimaki Y, Fujimori S, Izumida Y, Suzuki R, Ueki K, Kadowaki T, Hashimoto H (2016). A qualitative study on the impact of internalized stigma on type 2 diabetes self-management. Patient Educ Couns.

[9] World Health Organization (1998). Therapeutic patient education. Continuing education programme for healthcare providers in the field of prevention of chronic diseases.

[10] Bodenheimer T, Lorig K, Holman H, Grumbach K (2002). Patient self-management of chronic disease in primary care. JAMA.

[11] Barlow J, Wright C, Sheasby J, Turner A, Hainsworth J (2002). Self-management approaches for people with chronic conditions: a review. Patient Educ Couns.

[12] Lorig KR, Holman HR (2003). Self-management education: history, definition, outcomes, and mechanisms. Ann Behav Med.

[13] Stenberg U, Vågan A, Flink M, Lynggaard V, Fredriksen K, Westermann KF, Gallefoss F (2018). Health economic evaluations of patient education interventions a scoping review of the literature. Patient Educ Couns.

[14] Coster S, Norman I (2009). Cochrane reviews of educational and self-management interventions to guide nursing practice: a review. Int J Nurs Stud.

[15] Alvarez C, Greene J, Hibbard J, Overton V (2016). The role of primary care providers in patient activation and engagement in self-management: a cross-sectional analysis. BMC Health Serv Res.

[16] The Norwegian Directorate of Health (2016). Guidelines for Municipal Healthy Life Centers.

[17] Redman BK (2007). Responsibility for control; ethics of patient preparation for self-management of chronic disease. Bioethics.

[18] Bandura Albert (1977). Self-efficacy: Toward a unifying theory of behavioral change. Psychological Review.

[19] Thille P, Ward N, Russell G (2014). Self-management support in primary care: enactments, disruptions, and conversational consequences. Soc Sci Med.

[20] Vrangbaek K (2015). Patient involvement in Danish health care. J Health Organ Manag.

[21] Dent M, Pahor M (2015). Patient involvement in Europe–a comparative framework. J Health Organ Manag.

[22] Greenhalgh T (2009). Patient and public involvement in chronic illness: beyond the expert patient. Bmj.

[23] The Norwegian Directorate of Health (2019). Ledelse og kvalitetsforbedring i helse- og onsorgstjenesten- Nasjonal handlingsplan for pasientsikkerthet og kvalitetsforbedring 2019–2023 [National strategy for patient safety and quality improvements in health- and caring services].

[24] Beresford P, Carr S (2012). Social care, service users and user involvement.

[25] Barnes M, Cotterell P (2012). Critical perspectives on user involvement.

[26] The Norwegian Ministry of Health and Care Service (2013). NCD-strategy 2013–2017.

[27] Rollnick S, Miller WR (1995). What is motivational interviewing?. Behav Cogn Psychother.

[28] Miller WR, Rollnick S (2012). Motivational interviewing: helping people change.

[29] Markland D, Ryan RM, Tobin VJ, Rollnick S (2005). Motivational interviewing and self–determination theory. J Soc Clin Psychol.

[30] Sagsveen E, Rise MB, Grønning K, Westerlund H, Bratås O (2019). Respect, trust and continuity: a qualitative study exploring service users’ experience of involvement at a healthy life Centre in Norway. Health Expect.

[31] Salemonsen E, Førland G, Hansen BS, Holm AL (2019). Service users’ experience of beneficial self-management support and user involvement in healthy life Centres– a qualitative interview study submitted and under review.

[32] Følling IS, Solbjør M, Midthjell K, Kulseng B, Helvik A-S (2016). Exploring lifestyle and risk in preventing type 2 diabetes-a nested qualitative study of older participants in a lifestyle intervention program (VEND-RISK). BMC Public Health.

[33] Abildsnes E, Meland E, Samdal GB, Stea TH, Mildestvedt T (2016). Stakeholders’ expectations of healthy life centers: a focus group study. Scandinavian J Public Health.

[34] Sagsveen E, Rise MB, Grønning K, Bratås O (2018). Individual user involvement at healthy life Centres: a qualitative study exploring the perspective of health professionals. Int J Qual Stud Health Well Being.

[35] Polit DF, Beck CT (2017). Nursing Reseach - generating and assessing evidence for nursing practice.

[36] Vaismoradi M, Jones J, Turunen H, Snelgrove S (2016). Theme development in qualitative content analysis and thematic analysis. J Nurs Educ Pract.

[37] Kvale S, Brinkmann S (2015). Interviews. Learning the craft of qualitative research interviewing.

[38] Morgan DL. Focus Groups as Qualitative Research. 2nd ed. California: SAGE Publications Inc;1997.

[39] Malterud K, Siersma VD, Guassora AD (2016). Sample size in qualitative interview studies: guided by information power. Qual Health Res.

[40] Sandelowski M (1995). Sample size in qualitative research. Res Nurs Health.

[41] Morgan DL (1993). Successful focus groups: advancing the state of the art.

[42] Blaikie N (2009). Designing social research.

[43] Braun V, Clarke V (2006). Using thematic analysis in psychology. Qual Res Psychol.

[44] Krippendorff K (2013). Content analysis. An introduction to its methodology.

[45] Alvesson M, Sköldberg K (2018). Reflexive methodology: new vistas for qualitative research.

[46] Gadamer H-G, Weinsheimer J, Marshall DG (2013). Truth and method, 1st paperback ed. translation revised by Joel Weinsheimer and Donald G. Marshall. Edn.

[47] Sandelowski M, Leeman J (2012). Writing usable qualitative health research findings. Qual Health Res.

[48] Beauchamp TL, Childress JF. Principles of biomedical ethics. 7th ed. New York: Oxford Univeristy Press; 2012.

[49] Walseth LT, Abildsnes E, Schei E (2011). Patients’ experiences with lifestyle counselling in general practice: a qualitative study. Scand J Prim Health Care.

[50] Artinian NT, Fletcher GF, Mozaffarian D, Kris-Etherton P, Van Horn L, Lichtenstein AH, Kumanyika S, Kraus WE, Fleg JL, Redeker NS (2010). Interventions to promote physical activity and dietary lifestyle changes for cardiovascular risk factor reduction in adults. A scientific statement from the American Heart Association. Circulation.

[51] Svavarsdóttir MH, Sigurdardottir AK, Steinsbekk A (2016). What is a good educator? A qualitative study on the perspective of individuals with coronary heart disease. Eur J Cardiovasc Nurs.

[52] Gjengedal E, Ekra EM, Hol H, Kjelsvik M, Lykkeslet E, Michaelsen R, Orøy A, Skrondal T, Sundal H, Vatne S (2013). Vulnerability in health care–reflections on encounters in every day practice. Nurs Philos.

[53] Tranvåg O, Synnes O, McSherry W (2016). Stories of dignity within healthcare: research, narratives and theories.

[54] Benner P (1982). From novice to expert. AJN The American Journal of Nursing.

[55] Rogers CR (1981). The foundations of the person-centered approach. Dialectics and Humanism.

[56] Buber M. I and Thou. London: Bloomsbury Academic; 2013.

[57] Sikorski C, Luppa M, Glaesmer H, Brähler E, König H-H, Riedel-Heller SG (2013). Attitudes of health care professionals towards female obese patients. Obes Facts.

[58] Puhl RM, Heuer CA (2009). The stigma of obesity: a review and update. Obesity.

[59] Robstad N, Westergren T, Siebler F, Söderhamn U, Fegran L (2019). Intensive care nurses’ implicit and explicit attitudes and their behavioural intentions towards obese intensive care patients. J Adv Nurs.

[60] Malterud K, Ulriksen K (2011). Obesity, stigma, and responsibility in health care: a synthesis of qualitative studies. Int J Qual Stud Health Well Being.

[61] Franklin M, Lewis S, Willis K, Bourke-Taylor H, Smith L (2018). Patients’ and healthcare professionals’ perceptions of self-management support interactions: systematic review and qualitative synthesis. Chronic Illness.

[62] Svavarsdóttir MH, Sigurðardóttir ÁK, Steinsbekk A (2015). How to become an expert educator: a qualitative study on the view of health professionals with experience in patient education. BMC Med Educ.

[63] Middleton KR, Anton SD, Perri MG (2013). Long-term adherence to health behavior change. Am J Lifestyle Med.

[64] Greene J, Hibbard JH, Alvarez C, Overton V (2016). Supporting patient behavior change: approaches used by primary care clinicians whose patients have an increase in activation levels. Ann Fam Med.

[65] Ross Middleton K, Patidar S, Perri M (2012). The impact of extended care on the long-term maintenance of weight loss: a systematic review and meta-analysis. Obes Rev.

[66] Martins C: Wight loss maintenance - a tortuos path. Indremedisineren2018, https://indremedisinerenno/2018/02/weight-loss-maintenance-a-tortuous-path/ , , (04:2017).

[67] Perri MG, Ariel-Donges AH, Wadden TA, Bray GA (2018). Maintenance of weight lost in behavioral treatment of obesity. Handbook of Obesity Treatment.

[68] Morgan ML. The Cambridge introduction to Emmanuel Levinas. New York: Cambridge University Press; 2011.

[69] Levinas E (1995). Etik og uendelighed: samtaler med Phillipe Nemo [ethics and infinity conversations with Phillipe Nemo].

[70] Levinas E, Robbins J (2001). Is it righteous to be?: interviews with Emmanuel Levinas.

[71] Brownell KD (1991). Personal responsibility and control over our bodies: when expectation exceeds reality. Health Psychol.

[72] Brownell KD, Kersh R, Ludwig DS, Post RC, Puhl RM, Schwartz MB, Willett WC (2010). Personal responsibility and obesity: a constructive approach to a controversial issue. Health Aff.

[73] Thille P, Friedman M, Setchell J (2017). Weight-related stigma and health policy. CMAJ: Canadian Medical Association journal= journal de l'Association medicale canadienne.

[74] Malterud K (2010). Ulriksen K: “Norwegians fear fatness more than anything else”—a qualitative study of normative newspaper messages on obesity and health. Patient Educ Couns.

[75] Lincoln YS, Guba EG (1985). Naturalistic inquiry.

[76] Fleming V, Gaidys U, Robb Y (2003). Hermeneutic research in nursing: developing a Gadamerian-based research method. Nurs Inq.

[77] Tong A, Sainsbury P, Craig J (2007). Consolidated criteria for reporting qualitative research (COREQ): a 32-item checklist for interviews and focus groups. Int J Qual Health Care.

